# Predictive model for in-hospital acute cerebral infarction in patients with acute gastrointestinal bleeding: a retrospective cohort study

**DOI:** 10.3389/fmed.2026.1847050

**Published:** 2026-05-13

**Authors:** Yiqian Liang, Ling Zhang, Zunling Chen, Lingfeng Ruan, Tao Zhang

**Affiliations:** Department of Gastroenterology, The Affiliated Hospital of Southwest Medical University, Nanchong Central Hospital, Luzhou, Sichuan Province, China

**Keywords:** acute cerebral infarction, gastrointestinal bleeding, predictive model, PSM, risk factors

## Abstract

**Background:**

Acute gastrointestinal bleeding (GIB) is a common emergency in gastroenterology and may be accompanied by blood loss, anemia, systemic inflammation, and hemodynamic instability. Because the brain has a high metabolic demand and depends on continuous perfusion and oxygen delivery, patients with GIB may be vulnerable to in-hospital acute cerebral infarction (ACI). However, whether GIB is associated with a higher incidence of in-hospital ACI in gastroenterology inpatients remains insufficiently studied, and clinically practical risk stratification tools are lacking.

**Objective:**

To compare the incidence of in-hospital ACI between patients with and without GIB, identify factors associated with in-hospital ACI among patients with GIB, and develop a clinically applicable nomogram.

**Methods:**

A retrospective cohort study was conducted among gastroenterology inpatients at Nanchong Central Hospital between September 2020 and December 2025. Patients were classified as GIB or non-GIB. Propensity score matching (PSM) was used to compare in-hospital ACI incidence between groups. Among patients with GIB, candidate predictors were evaluated using univariable and multivariable logistic regression, and a nomogram was developed and internally validated. Sensitivity, alternative, and exploratory subgroup analyses were additionally performed.

**Results:**

A total of 2,734 patients with suspected GIB and 10,763 patients without GIB were initially screened; after exclusion, 2,380 and 8,996 patients, respectively, were included. After 1:1 PSM, 2,380 matched pairs were analyzed. In-hospital ACI occurred more frequently in the GIB group than in the non-GIB group (7.8% vs. 5.2%, *p* < 0.001). Among patients with GIB, previous cerebral infarction history (OR 13.47, 95% CI 9.26–19.60), sepsis and infection (OR 2.43, 95% CI 1.71–3.44), cerebral hemorrhage (OR 3.48, 95% CI 1.17–10.33), anemia (OR 1.54, 95% CI 1.02–2.32), age (OR 1.05 per year, 95% CI 1.03–1.07), and length of hospital stay (OR 1.04 per day, 95% CI 1.02–1.07) were independently associated with in-hospital ACI. The nomogram demonstrated good discrimination (AUC 0.864, 95% CI 0.837–0.888) and calibration (MAE 0.008).

**Conclusion:**

In this single-center retrospective cohort, GIB was associated with a higher incidence of in-hospital ACI. A six-factor nomogram based on routinely available clinical variables showed good internal performance and may assist risk stratification, although external validation is needed before broader clinical use.

## Introduction

Gastrointestinal bleeding refers to bleeding from the esophagus to the anus and may be classified as upper, small-bowel, or lower gastrointestinal bleeding, with upper gastrointestinal bleeding being the most common form in clinical practice (1). The brain has high metabolic demand and is highly dependent on continuous cerebral blood flow and oxygen delivery (2). Acute blood loss, anemia, systemic inflammation, and hemodynamic instability may therefore create a biological context in which cerebral ischemia becomes more likely, particularly in patients with pre-existing cerebrovascular vulnerability (3–6). At the same time, stroke remains a major cause of death and disability worldwide (7). Despite these considerations, direct evidence addressing whether GIB is associated with a higher incidence of in-hospital acute cerebral infarction in gastroenterology inpatients remains limited. In addition, targeted bedside tools for identifying high-risk GIB patients are lacking. Accordingly, this study compared in-hospital ACI incidence between patients with and without GIB after propensity score matching, identified factors independently associated with in-hospital ACI among patients with GIB, and constructed a nomogram for individualized risk estimation.

## Materials and methods

### Study design and patients

This was a retrospective cohort study of adult patients admitted to the Department of Gastroenterology, Nanchong Central Hospital, between September 2020 and December 2025. Patients were classified into an exposed group (with gastrointestinal bleeding) and a control group (without gastrointestinal bleeding) according to the occurrence of GIB during hospitalization. After application of predefined exclusion criteria, 2,380 patients with GIB and 8,996 patients without GIB were included in the cohort analysis. All outcomes were assessed during the index hospitalization.

Inclusion criteria for the exposed group: 1. Age ≥ 18 years; 2. Clinical manifestations such as hematemesis, coffee-ground vomitus, melena, hematochezia, hemorrhagic peripheral circulatory failure, or strongly positive fecal occult blood test, confirmed by clinical manifestations, electronic endoscopy, or laboratory examinations.

Inclusion criteria for the control group: 1. Exclusion of endoscopic, laboratory, and clinical evidence of gastrointestinal bleeding; 2. Common non-bleeding diseases including but not limited to: functional gastrointestinal disorders, reflux esophagitis, chronic non-atrophic gastritis, *Helicobacter pylori*-associated gastritis, gastric ulcer; colonic polyps, chronic enteritis, Crohn’s disease in remission, ulcerative colitis in remission; chronic cholecystitis, gallstones; 3. Completion of the same basic examinations as the exposed group during hospitalization, such as blood routine, coagulation function, liver and kidney function, cranial CT/MRI, electrocardiogram, etc.

Inclusion criteria for patients with acute in-hospital cerebral infarction: 1. Age ≥ 18 years, hospitalized in the Department of Gastroenterology, Nanchong Central Hospital, from September 2020 to December 2025, with new-onset acute cerebral infarction symptoms or confirmed by examinations during hospitalization; 2. New cerebral infarction lesions confirmed by CT or MRI; 3. Clinical manifestations: acute onset of focal neurological deficit symptoms, including but not limited to: hemiplegia or limb weakness, hemisensory disturbance, speech disorder, binocular gaze disturbance, dysphagia, ataxia, etc.; symptom duration is not limited; patients with imaging-confirmed responsible ischemic lesions and symptom duration <24 h are included; in the absence of imaging evidence, symptoms must last ≥24 h; 4. Confirmed diagnosis of acute ischemic stroke by neurology consultation based on medical history, physical examination, imaging results, and laboratory examinations, excluding traumatic brain injury, poisoning, and other causes.

Exclusion criteria: (1) voluntary discharge during hospitalization or refusal to cooperate with treatment; (2) incomplete medical records or missing key laboratory/clinical data; (3) end-stage diseases, including severe liver or renal insufficiency, advanced malignant tumors, or severe coagulation disorders; and (4) cases with secondary gastrointestinal bleeding or unconfirmed bleeding status during screening.

### Methods

This was a retrospective observational study based on routinely collected clinical data. Variables extracted from the medical record included sex, age, length of hospital stay, hypertension, diabetes mellitus, coronary heart disease, previous cerebral infarction history, cerebral hemorrhage, admission hemoglobin level, sepsis and infection, renal insufficiency, liver insufficiency, use of octreotide and somatostatin, surgical or endoscopic hemostatic therapy, anemia, bleeding etiology, shock, and cerebrovascular disease. These variables were used for cohort comparison, risk factor identification within the GIB cohort, and model development.

### Statistical analysis

Propensity Score Matching (PSM): To reduce baseline imbalance between patients with and without GIB, propensity scores were estimated using age, sex, hypertension, diabetes mellitus, coronary heart disease, previous cerebral infarction history, and cerebral hemorrhage. A 1:1 nearest-neighbor matching strategy with a caliper of 0.25 was applied. Baseline characteristics were then compared in the matched cohort to assess post-matching balance.

Risk Factor Analysis: Within the GIB cohort, in-hospital ACI was used as the dependent variable. Candidate variables were first evaluated using descriptive comparison and univariable logistic regression. Variables with *p* < 0.05 were entered into a multivariable logistic regression model, and backward stepwise selection was used to identify factors independently associated with in-hospital ACI. Results are presented as odds ratios (ORs) with 95% confidence intervals (CIs).

Construction and Validation of Predictive Model: A nomogram was constructed on the basis of the final multivariable logistic regression model. Model discrimination was assessed using the receiver operating characteristic (ROC) curve and the area under the curve (AUC), and calibration was assessed using calibration plots and the mean absolute error (MAE) between predicted and observed event probabilities. Internal validation was performed by bootstrap resampling (1,000 repetitions). Additional analyses included a sensitivity model excluding length of hospital stay, an alternative model replacing anemia with continuous hemoglobin level, and exploratory subgroup analyses; detailed results are presented in Supplementary material.

## Results

### Patient selection and study flow

**Figure 1:**
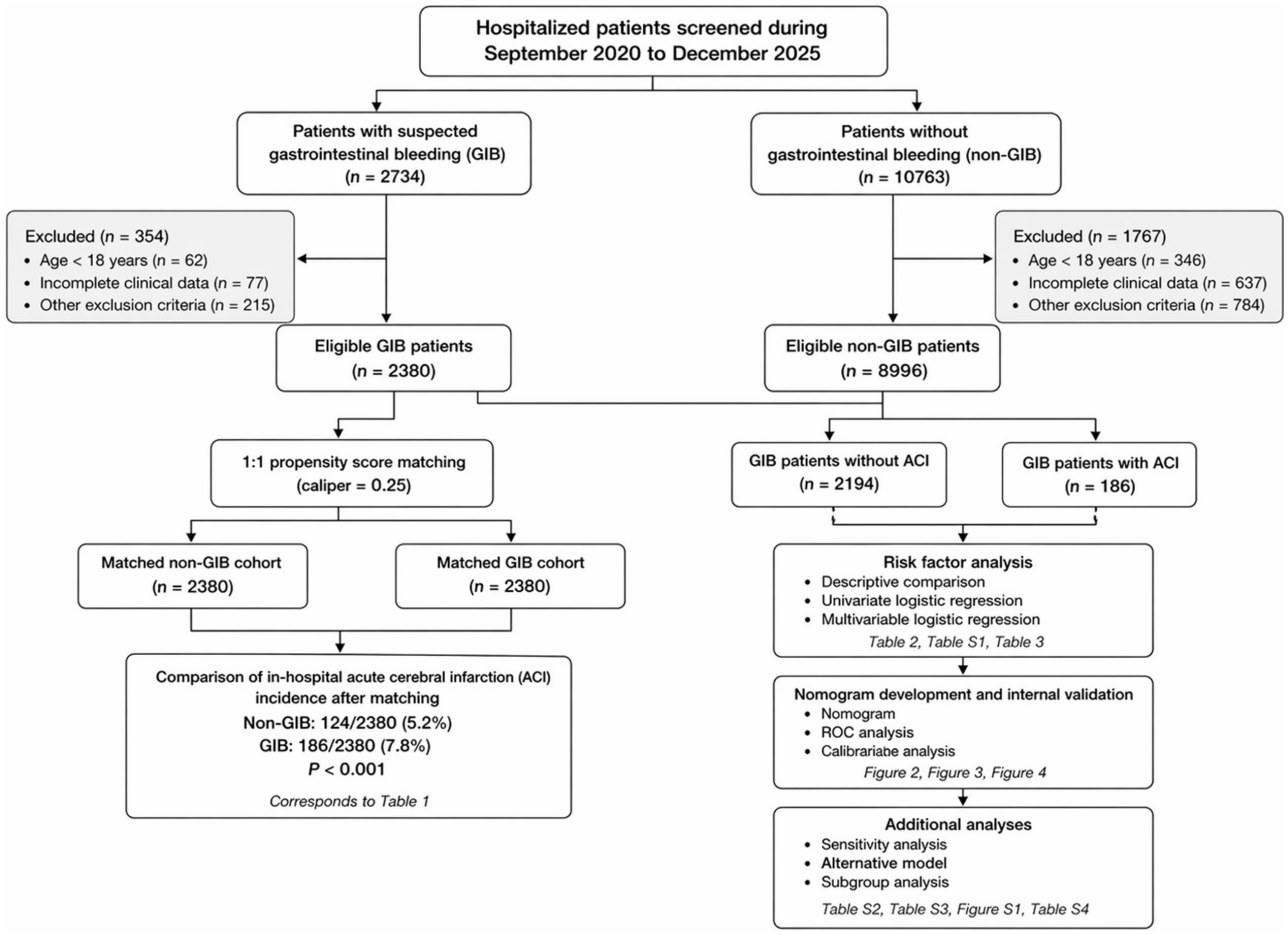
Study flow diagram. Flowchart of patient screening, exclusion, cohort allocation, propensity score matching, and downstream analyses. A total of 2,734 patients with suspected gastrointestinal bleeding (GIB) and 10,763 patients without GIB were initially screened. After exclusion, 2,380 patients with GIB and 8,996 patients without GIB were included in the cohort analysis. After 1:1 propensity score matching, 2,380 matched pairs were analyzed. Within the GIB cohort, 186 patients developed in-hospital acute cerebral infarction (ACI) and 2,194 did not. GIB, gastrointestinal bleeding; ACI, acute cerebral infarction; PSM, propensity score matching.

During the study period, 2,734 patients with suspected GIB and 10,763 patients without GIB were initially screened. After exclusion of cases with end-stage disease, secondary gastrointestinal bleeding, unconfirmed bleeding status, or missing data, 2,380 patients with GIB and 8,996 patients without GIB remained eligible for analysis. Within the GIB cohort, 186 patients developed in-hospital ACI and 2,194 did not. The overall study flow, exclusions, matching strategy, and downstream analyses are summarized in Figure 1.

### Matched baseline characteristics and in-hospital ACI incidence after PSM

After 1:1 PSM, 2,380 matched pairs were generated. As summarized in Tables 1, 2, age, sex, hypertension, diabetes mellitus, coronary heart disease, previous cerebral infarction history, and cerebral hemorrhage were well balanced between the two groups after matching (all *p* > 0.05), indicating satisfactory post-matching comparability.

**Table 1:** Baseline characteristics of the matched cohort after propensity score matching.

Variables	Non-gastrointestinal bleeding group (*n* = 2,380)	Gastrointestinal bleeding group (*n* = 2,380)	Statistical value	*p* value
Length of hospital stay	9.97 ± 6.91	9.72 ± 6.37	1.279	0.201
Age (years)	62.38 ± 16.19	63.11 ± 15.72	−1.573	0.116
Gender			0.132	0.716
Male	1,544 (64.9%)	1,532 (64.4%)		
Female	836 (35.1%)	848 (35.6%)		
Hypertension	719 (30.2%)	740 (31.1%)	0.436	0.509
Diabetes mellitus	412 (17.3%)	415 (17.4%)	0.013	0.909
Coronary heart disease	226 (9.5%)	218 (9.2%)	0.159	0.690
Previous cerebral infarction history	155 (6.5%)	185 (7.8%)	2.851	0.091
Cerebral hemorrhage	13 (0.5%)	25 (1.1%)	3.820	0.051

**Table 2:** Comparison of in-hospital acute cerebral infarction incidence after propensity score matching.

Cerebral infarction	Non-gastrointestinal bleeding group (*n* = 2,380)	Gastrointestinal bleeding group (*n* = 2,380)	*χ*^2^	*p* value
No	2,256 (94.8%)	2,194 (92.2%)	13.264	<0.001
Yes	124 (5.2%)	186 (7.8%)		

Incidence of in-hospital ACI after PSM.

In the matched cohort of 4,760 patients, in-hospital ACI occurred in 186 of 2,380 patients (7.8%) in the GIB group and in 124 of 2,380 patients (5.2%) in the non-GIB group. This difference was statistically significant (*χ*^2^ = 13.264, *p* < 0.001), indicating that GIB was associated with a higher in-hospital incidence of ACI after matching.

### Descriptive comparison between ACI and non-ACI in patients with GIB

Among 2,380 patients with GIB, 186 (7.82%) developed in-hospital ACI and 2,194 (92.18%) did not. Compared with the non-ACI subgroup, patients with ACI were older, had longer hospital stays and lower hemoglobin levels, and more frequently had hypertension, diabetes mellitus, coronary heart disease, sepsis and infection, renal insufficiency, previous cerebral infarction history, anemia, cerebral hemorrhage, and cerebrovascular disease. They were also less likely to have undergone surgical or endoscopic hemostatic therapy and less likely to have esophageal or gastric variceal bleeding. No significant differences were observed for male sex, liver insufficiency, use of octreotide and somatostatin, ulcer-related bleeding, cancer-related bleeding, or shock (Table 3). The full univariable logistic regression results for all candidate variables are provided in Supplementary Table S1.

**Table 3:** Comparison of clinical characteristics between patients with and without in-hospital acute cerebral infarction among gastrointestinal bleeding patients.

Variable	No acute cerebral infarction (*n* = 2,194)	Acute cerebral infarction (*n* = 186)	Statistic	*p* value
Length of hospital stay	8.00 (5.00, 12.00)	10.00 (7.00, 16.00)	*U* = 153,519.00	<0.001
Age	64.00 (52.00, 74.00)	74.00 (68.00, 81.00)	*U* = 115,109.00	<0.001
Hemoglobin level	99.00 (70.00, 129.00)	88.00 (70.00, 114.75)	*U* = 228,416.00	0.007
Male sex	1,414 (64.45%)	118 (63.44%)	*χ*^2^ = 0.08	0.783
Hypertension	641 (29.22%)	99 (53.23%)	*χ*^2^ = 46.13	<0.001
Diabetes mellitus	362 (16.50%)	53 (28.49%)	*χ*^2^ = 17.14	<0.001
Coronary heart disease	189 (8.61%)	29 (15.59%)	*χ*^2^ = 10.03	0.002
Sepsis and infection	771 (35.14%)	107 (57.53%)	*χ*^2^ = 36.91	<0.001
Renal insufficiency	204 (9.30%)	33 (17.74%)	*χ*^2^ = 13.63	<0.001
Liver insufficiency	308 (14.04%)	29 (15.59%)	*χ*^2^ = 0.34	0.560
Previous cerebral infarction history	101 (4.60%)	84 (45.16%)	*χ*^2^ = 393.43	<0.001
Use of octreotide and somatostatin	709 (32.32%)	55 (29.57%)	*χ*^2^ = 0.59	0.441
Surgical or endoscopic hemostatic therapy	397 (18.09%)	14 (7.53%)	*χ*^2^ = 13.40	<0.001
Anemia	1,361 (62.03%)	146 (78.49%)	*χ*^2^ = 20.01	<0.001
Ulcer-related bleeding	633 (28.85%)	57 (30.65%)	*χ*^2^ = 0.27	0.605
Esophageal and gastric variceal bleeding	230 (10.48%)	6 (3.23%)	*χ*^2^ = 10.11	0.001
Cancer-related bleeding	98 (4.47%)	5 (2.69%)	*χ*^2^ = 1.31	0.252
Cerebral hemorrhage	19 (0.87%)	6 (3.23%)	*χ*^2^ = 9.19	0.002
Cerebrovascular disease	5 (0.23%)	3 (1.61%)	Fisher	0.020
Shock	199 (9.07%)	22 (11.83%)	*χ*^2^ = 1.55	0.213

Continuous variables are presented as median (Q1, Q3) and were compared using the Mann–Whitney U test. Categorical variables are presented as n (%) and were compared using the *χ*^2^ test or Fisher’s exact test, as appropriate. ACI, acute cerebral infarction.

### Multivariable logistic regression and nomogram development

**Figure 2:**
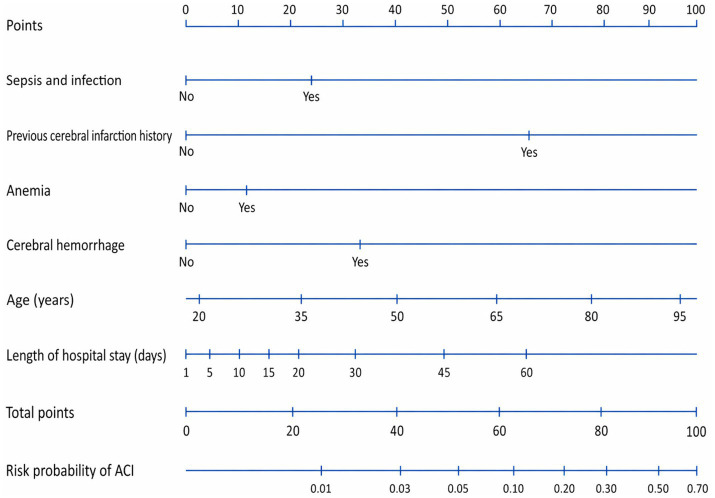
Nomogram for individualized prediction of in-hospital acute cerebral infarction in patients with gastrointestinal bleeding. The nomogram was developed from the final multivariable logistic regression model and includes length of hospital stay, age, sepsis and infection, previous cerebral infarction history, anemia, and cerebral hemorrhage. For each predictor, the corresponding score is assigned on the upper scale, and the total score corresponds to the estimated probability of in-hospital ACI on the lower scale. ACI, acute cerebral infarction.

Variables with *p* < 0.05 in univariable analysis were entered into the multivariable logistic regression model. Length of hospital stay (OR 1.04, 95% CI 1.02–1.07, *p* < 0.001), age (OR 1.05, 95% CI 1.03–1.07, *p* < 0.001), sepsis and infection (OR 2.43, 95% CI 1.71–3.44, *p* < 0.001), previous cerebral infarction history (OR 13.47, 95% CI 9.26–19.60, *p* < 0.001), anemia (OR 1.54, 95% CI 1.02–2.32, *p* = 0.039), and cerebral hemorrhage (OR 3.48, 95% CI 1.17–10.33, *p* = 0.025) remained independently associated with in-hospital ACI in patients with GIB (Table 4). Based on these six variables, a nomogram was constructed for individualized risk estimation (Figure 2).

**Table 4:** Multivariable logistic regression analysis of factors associated with in-hospital acute cerebral infarction in gastrointestinal bleeding patients.

Variable	*β*	SE	*Z*	*p* value	OR (95% CI)
Length of hospital stay	0.0423	0.0113	3.751	<0.001	1.04 (1.02–1.07)
Age	0.0485	0.0074	6.523	<0.001	1.05 (1.03–1.07)
Sepsis and infection	0.8878	0.1779	4.989	<0.001	2.43 (1.71–3.44)
Previous cerebral infarction history	2.6006	0.1914	13.589	<0.001	13.47 (9.26–19.60)
Anemia	0.4310	0.2088	2.064	0.039	1.54 (1.02–2.32)
Cerebral hemorrhage	1.2475	0.5549	2.248	0.025	3.48 (1.17–10.33)

*β*, regression coefficient; SE, standard error; OR, odds ratio; CI, confidence interval. Variables entered into the final multivariable model are shown.

### Model discrimination and calibration

**Figure 3:**
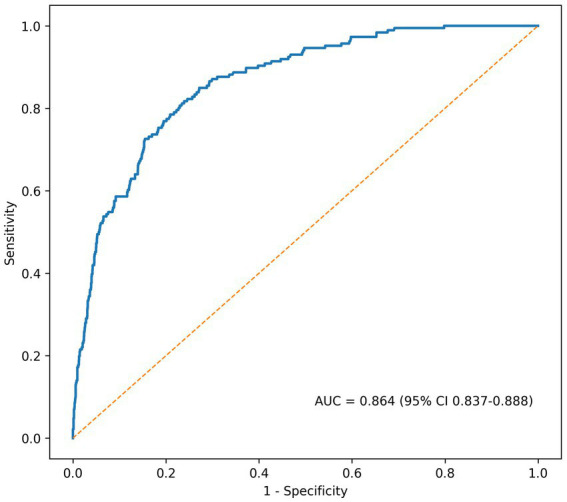
Receiver operating characteristic curve of the nomogram. The nomogram demonstrated good discrimination for predicting in-hospital acute cerebral infarction in patients with gastrointestinal bleeding, with an area under the curve (AUC) of 0.864 (95% CI 0.837–0.888). AUC, area under the curve; CI, confidence interval; ACI, acute cerebral infarction.

**Figure 4:**
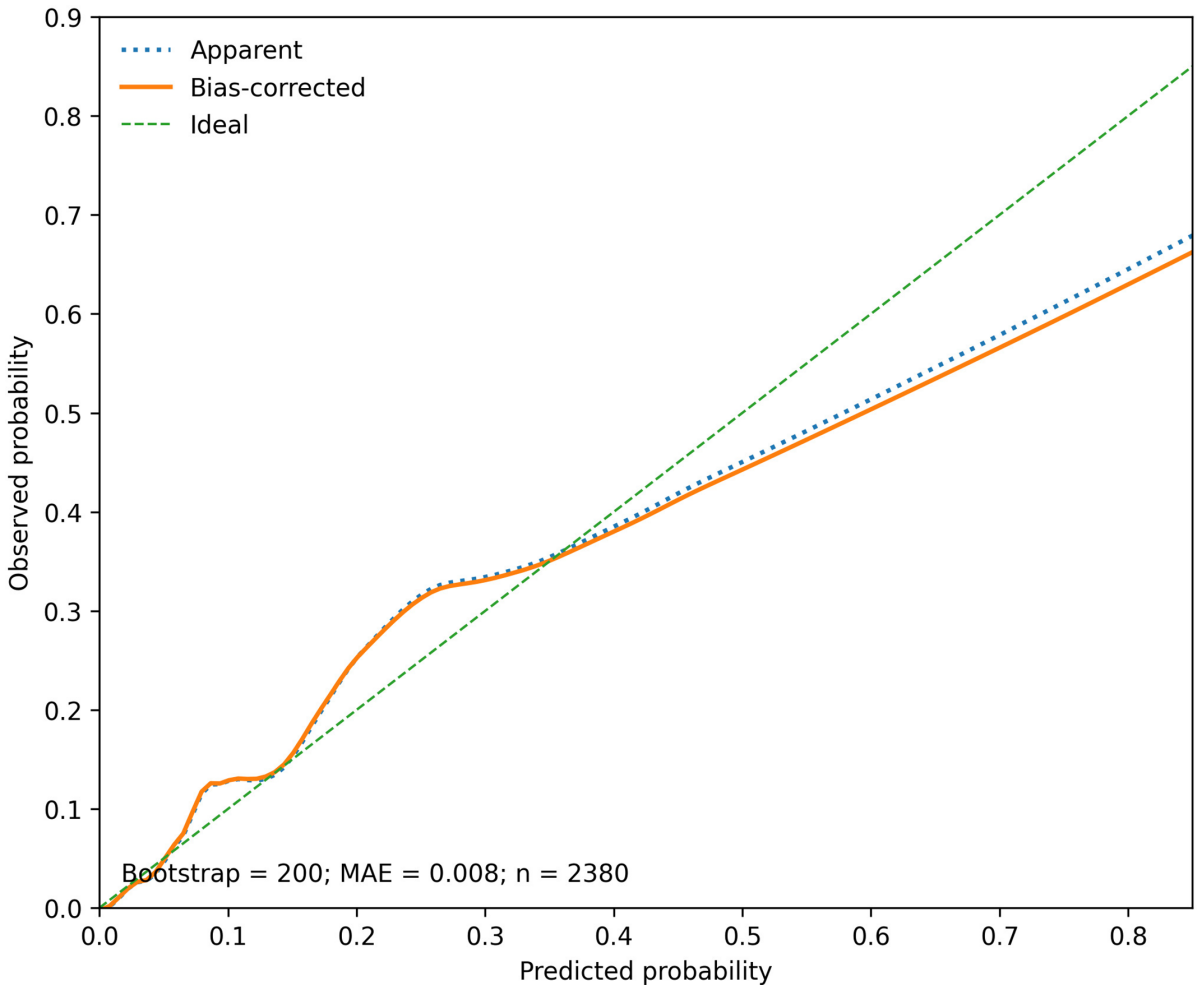
Calibration plot of the nomogram. Calibration analysis showed good agreement between predicted and observed probabilities of in-hospital acute cerebral infarction, with a mean absolute error (MAE) of 0.008. The model was internally validated by bootstrap resampling. MAE, mean absolute error; ACI, acute cerebral infarction.

The nomogram derived from the final multivariable model is shown in Figure 2. On ROC analysis, the model demonstrated good discrimination, with an AUC of 0.864 (95% CI 0.837–0.888) (Figure 3). Calibration was also good, with close agreement between predicted and observed probabilities and a mean absolute error of 0.008 (Figure 4).

### Sensitivity, alternative, and subgroup analyses

Sensitivity analysis excluding length of hospital stay yielded a five-factor model based on age, sepsis and infection, previous cerebral infarction history, anemia, and cerebral hemorrhage, with only a slight reduction in discrimination (AUC 0.858 vs. 0.864 in the main model). In alternative modeling, substituting continuous hemoglobin for anemia produced similar but slightly weaker performance (AUC 0.860), and hemoglobin was not independently associated after adjustment. Exploratory subgroup analyses suggested particularly high absolute and relative risks among patients aged ≥70 years, those with previous cerebral infarction history, and those with sepsis/infection, whereas variceal bleeding was associated with a lower unadjusted incidence of in-hospital ACI. Detailed results are provided in Supplementary Tables S2A–S4B and Supplementary Figure S1.

## Discussion

This study addressed a clinically relevant but understudied question in gastroenterology inpatients. Using a matched cohort comparison followed by within-GIB modeling, we found that GIB was associated with a higher in-hospital incidence of ACI, and that previous cerebral infarction history, sepsis and infection, cerebral hemorrhage, anemia, age, and length of hospital stay were independently associated with in-hospital ACI among patients with GIB. Based on these variables, we developed a nomogram that showed good discrimination and calibration.

After 1:1 PSM, the incidence of in-hospital ACI remained higher in the GIB group than in the non-GIB group (7.8% vs. 5.2%). Because this study was retrospective and observational, these results should be interpreted as an association rather than proof that GIB itself causes ACI. Nevertheless, the finding is biologically plausible: acute bleeding may reduce oxygen-carrying capacity and circulating volume, while systemic stress and inflammatory activation may further impair cerebrovascular reserve in susceptible patients (3–6, 8).

Previous cerebral infarction history was the strongest factor associated with in-hospital ACI in the multivariable model (OR 13.47, 95% CI 9.26–19.60). This likely reflects pre-existing cerebrovascular vulnerability, including impaired autoregulatory reserve, residual arterial disease, and a lower threshold for ischemic decompensation during acute physiological stress (2, 6, 9). Age was also independently associated with ACI, with the odds increasing by about 5% per year. This is consistent with the cumulative burden of vascular stiffness, multimorbidity, and reduced physiological reserve in older patients. In the exploratory subgroup analyses, patients aged ≥70 years showed substantially higher absolute risk, reinforcing the clinical importance of age-sensitive surveillance.

Sepsis and infection were also independently associated with in-hospital ACI (OR 2.43, 95% CI 1.71–3.44). Systemic infection may increase stroke vulnerability through endothelial dysfunction, inflammatory cytokine activation, coagulation abnormalities, and immunothrombotic pathways (10–14). Clinically, this finding supports heightened attention to early identification and control of infection in patients with GIB, particularly when other high-risk features coexist.

Cerebral hemorrhage remained independently associated with in-hospital ACI (OR 3.48, 95% CI 1.17–10.33). Although the absolute number of such patients was small, this association may reflect a fragile cerebrovascular substrate in which hemorrhagic and ischemic mechanisms coexist, together with therapeutic complexity related to blood pressure control, antithrombotic exposure, and cerebral perfusion management (15). Anemia was also independently associated with ACI (OR 1.54, 95% CI 1.02–2.32). This result is directionally consistent with prior literature indicating that impaired oxygen-carrying status is linked to worse ischemic stroke biology and outcomes (3, 16).

Importantly, the supplementary analyses strengthened the clinical interpretation of the main model. When length of hospital stay was removed, model discrimination declined only slightly (AUC 0.858 vs. 0.864), suggesting that a model based only on variables available earlier during hospitalization may still retain practical value. Likewise, replacing anemia with continuous hemoglobin level produced similar but slightly weaker performance, and hemoglobin itself was not independently associated after adjustment. Together, these findings suggest that a threshold-based anemia variable may provide a more stable and clinically interpretable representation of oxygen-carrying impairment in this setting.

Length of hospital stay was independently associated with ACI in the main model, but this variable should be interpreted cautiously. LOS may partly reflect greater illness complexity, in-hospital complications, or delayed recovery rather than a pure baseline predictor. For that reason, we examined a reduced model without LOS and found only modest attenuation of performance. In exploratory subgroup analyses, the highest-risk groups were patients with previous cerebral infarction history, older age, and sepsis/infection, whereas variceal bleeding showed a lower unadjusted incidence of ACI. These subgroup findings are hypothesis-generating and may help guide future risk-focused studies.

Several conventional vascular comorbidities, including hypertension, diabetes mellitus, coronary heart disease, and renal insufficiency, were associated with ACI in descriptive or univariable analyses but did not remain independently associated after multivariable adjustment. This does not mean that these conditions, particularly diabetes mellitus, are clinically unimportant, as diabetes is an established risk factor for ischemic stroke in the general population (17). This does not mean that these conditions are clinically unimportant. Rather, within the specific context of GIB, their effects may be partly mediated or overshadowed by more proximal factors such as previous cerebral infarction history, acute infection, anemia, and overall illness burden. Similarly, surgical or endoscopic hemostatic therapy was not independently associated with ACI after adjustment, suggesting that bleeding-control procedures themselves should not be interpreted as either protective or harmful with respect to ACI risk in this dataset.

The nomogram demonstrated good internal performance, with an AUC of 0.864 and good calibration (MAE 0.008). A practical strength of this model is that its predictors are routinely available in daily inpatient care, which supports bedside applicability. In addition, the supplementary sensitivity and alternative analyses showed that the model structure was reasonably robust. Compared with previously reported prediction work in upper gastrointestinal bleeding populations (18), the present model focuses on a broader gastroenterology inpatient cohort and emphasizes clinically accessible variables rather than highly specialized measurements. Such a tool may help clinicians identify patients who warrant closer neurological monitoring and more aggressive management of modifiable contributors such as infection and anemia.

This study has several limitations. First, it was a single-center retrospective analysis and is therefore subject to selection bias, information bias, and residual confounding. Important factors such as bleeding severity, transfusion strategy, antithrombotic exposure, and time-varying physiological changes were not fully captured. Second, internal validation was performed, but external validation was not available. Third, some subgroup analyses involved relatively small samples and should be regarded as exploratory. Accordingly, the model should be interpreted as a hypothesis-supporting and risk-stratification tool rather than a definitive causal framework.

## Conclusion

In this single-center retrospective cohort, GIB was associated with a higher in-hospital incidence of ACI than non-GIB inpatients after propensity score matching. Within the GIB cohort, previous cerebral infarction history, sepsis and infection, cerebral hemorrhage, anemia, age, and length of hospital stay were independently associated with in-hospital ACI. The derived nomogram showed good internal discrimination and calibration and may support early identification of high-risk patients. However, external validation and multicenter prospective studies are needed before routine clinical implementation.

## Data Availability

The data analyzed in this study is subject to the following licenses/restrictions: the raw clinical data used in this study are derived from the hospital’s internal electronic medical record system and contain protected patient health information. Due to ethical restrictions and patient privacy requirements, these datasets are not publicly available. Requests to access de-identified, aggregate-level data for reasonable purposes may be submitted to the corresponding author, subject to institutional review board approval and data use agreements. Requests to access these datasets should be directed to 305514271@qq.com.
