# Supplementary material for: Predictive model for in-hospital acute cerebral infarction in patients with acute gastrointestinal bleeding: a retrospective cohort study
Source: Front Med (Lausanne). 2026 May 13;13:1847050. doi: 10.3389/fmed.2026.1847050 (PMC13212107; doi:10.3389/fmed.2026.1847050)
Supplement: Supplementary file 1 [file Table_1.docx]

**Supplementary Material**

This Supplementary Material provides additional methodological details and supplementary results for the robustness analyses of the primary model, including sensitivity analysis, alternative modeling strategies, and exploratory subgroup analyses.

Supplementary Methods

S1. Sensitivity analysis excluding length of hospital stay

To evaluate whether the primary model remained clinically applicable when restricted to variables potentially available earlier during hospitalization, a sensitivity analysis was performed by excluding length of hospital stay from the multivariable logistic regression model. The rationale for this analysis was that length of hospital stay, although statistically significant in the primary model, may partly reflect post-admission disease evolution rather than a variable available at the time of early risk assessment. Therefore, a reduced model was constructed using age, sepsis and infection, previous cerebral infarction history, anemia, and cerebral hemorrhage, while all other modeling procedures were kept consistent with those of the primary analysis.

The discrimination and calibration of the reduced model were then compared with those of the primary model. Discrimination was assessed using the area under the receiver operating characteristic curve (AUC), and calibration was summarized using the mean absolute error (MAE) between predicted and observed event probabilities across deciles of risk. Changes in regression coefficients, odds ratios, and model performance metrics were examined to determine whether exclusion of length of hospital stay materially altered the predictive performance or interpretation of the model.

S2. Alternative modeling strategy using anemia versus hemoglobin

Because both anemia and hemoglobin level may reflect impaired oxygen-carrying status, an alternative modeling analysis was conducted to examine the robustness of this association. In the primary multivariable model, anemia was retained as a binary clinical variable, whereas hemoglobin level was significant only in univariate analysis. To further compare these two approaches, two alternative multivariable models were constructed: one model retained anemia as a predictor, and the other replaced anemia with continuous hemoglobin level.

The purpose of this analysis was to determine whether the association between impaired oxygen transport and in-hospital acute cerebral infarction remained directionally stable under different variable definitions, and to assess which form provided better model stability and clinical interpretability. The two models were compared in terms of regression coefficients, odds ratios, AUC, and MAE.

S3. Exploratory subgroup analyses

Exploratory subgroup analyses were performed to further assess whether the risk of in-hospital acute cerebral infarction varied across clinically relevant patient subsets. Prespecified subgroup variables included age, previous cerebral infarction history, sepsis and infection status, ulcer-related bleeding, esophageal/gastric variceal bleeding, and cancer-related bleeding. For each subgroup, the exposed category was compared with the corresponding reference category using unadjusted event counts, and odds ratios with 95% confidence intervals were calculated from 2×2 contingency tables. Fisher’s exact test was used when appropriate.

In addition, the discrimination of the primary multivariable model was explored within each subgroup by applying the primary model to subgroup-specific data and calculating the AUC separately. These subgroup analyses were intended to provide descriptive and exploratory information regarding risk heterogeneity and subgroup-specific model performance, rather than to establish formal interaction effects.

**Table S1. Univariate logistic regression analysis of factors associated with in-hospital acute cerebral infarction in gastrointestinal bleeding patients**

| **Variable** | **β** | **SE** | **OR (95% CI)** | **P value** |
| --- | --- | --- | --- | --- |
| Length of hospital stay | 0.0598 | 0.0094 | 1.06 (1.04-1.08) | <0.001 |
| Age | 0.0602 | 0.0068 | 1.06 (1.05-1.08) | <0.001 |
| Hemoglobin level | -0.0061 | 0.0022 | 0.99 (0.99-1.00) | 0.006 |
| Male sex | -0.0433 | 0.1570 | 0.96 (0.71-1.31) | 0.783 |
| Hypertension | 1.0141 | 0.1544 | 2.76 (2.04-3.73) | <0.001 |
| Diabetes mellitus | 0.7015 | 0.1745 | 2.02 (1.44-2.83) | <0.001 |
| Coronary heart disease | 0.6727 | 0.2161 | 1.96 (1.28-2.99) | 0.002 |
| Sepsis and infection | 0.9162 | 0.1541 | 2.50 (1.85-3.39) | <0.001 |
| Renal insufficiency | 0.7438 | 0.2054 | 2.10 (1.41-3.15) | <0.001 |
| Liver insufficiency | 0.1237 | 0.2114 | 1.13 (0.75-1.72) | 0.560 |
| Previous cerebral infarction history | 2.8371 | 0.1787 | 17.07 (12.01-24.24) | <0.001 |
| Use of octreotide and somatostatin | -0.0838 | 0.1092 | 0.92 (0.74-1.14) | 0.441 |
| Surgical or endoscopic hemostatic therapy | -0.9985 | 0.2814 | 0.37 (0.21-0.64) | <0.001 |
| Anemia | 0.8038 | 0.1842 | 2.23 (1.56-3.20) | <0.001 |
| Ulcer-related bleeding | 0.0866 | 0.1677 | 1.09 (0.78-1.50) | 0.605 |
| Esophageal and gastric variceal bleeding | -1.2565 | 0.4219 | 0.28 (0.12-0.65) | 0.003 |
| Cancer-related bleeding | -0.5206 | 0.4584 | 0.59 (0.24-1.45) | 0.252 |
| Cerebral hemorrhage | 1.3391 | 0.4743 | 3.82 (1.50-9.67) | 0.005 |
| Cerebrovascular disease | 1.9709 | 0.7343 | 7.18 (1.70-30.27) | 0.007 |
| Shock | 0.3047 | 0.2448 | 1.36 (0.84-2.19) | 0.213 |

*Note: Each candidate variable was entered into a separate univariate logistic regression model. β, regression coefficient; SE, standard error; OR, odds ratio; CI, confidence interval.*

**Supplementary Table S2A. Coefficients of the main model and the sensitivity model excluding length of hospital stay**

| **Model** | **Variable** | **β** | **SE** | **OR (95% CI)** | **P value** |
| --- | --- | --- | --- | --- | --- |
| Model 1: Main model | Length of hospital stay | 0.0423 | 0.0113 | 1.04 (1.02-1.07) | <0.001 |
| Model 1: Main model | Age | 0.0485 | 0.0074 | 1.05 (1.03-1.07) | <0.001 |
| Model 1: Main model | Sepsis and infection | 0.8878 | 0.1779 | 2.43 (1.71-3.44) | <0.001 |
| Model 1: Main model | Previous cerebral infarction history | 2.6006 | 0.1914 | 13.47 (9.26-19.60) | <0.001 |
| Model 1: Main model | Anemia | 0.431 | 0.2088 | 1.54 (1.02-2.32) | 0.039 |
| Model 1: Main model | Cerebral hemorrhage | 1.2475 | 0.5549 | 3.48 (1.17-10.33) | 0.025 |
| Model 2: Sensitivity model without LOS | Age | 0.0502 | 0.0074 | 1.05 (1.04-1.07) | <0.001 |
| Model 2: Sensitivity model without LOS | Sepsis and infection | 0.9576 | 0.1761 | 2.61 (1.84-3.68) | <0.001 |
| Model 2: Sensitivity model without LOS | Previous cerebral infarction history | 2.5868 | 0.1897 | 13.29 (9.16-19.27) | <0.001 |
| Model 2: Sensitivity model without LOS | Anemia | 0.5102 | 0.2065 | 1.67 (1.11-2.50) | 0.013 |
| Model 2: Sensitivity model without LOS | Cerebral hemorrhage | 1.1192 | 0.5468 | 3.06 (1.05-8.94) | 0.041 |

*Note: Model 1 is the primary model including length of hospital stay. Model 2 excludes length of hospital stay to evaluate whether the model remains stable when restricted to variables potentially available earlier during hospitalization. β, regression coefficient; SE, standard error; OR, odds ratio; CI, confidence interval; LOS, length of stay.*

**Supplementary Table S2B. Performance comparison between the main model and the sensitivity model excluding length of hospital stay**

| **Model** | **Variables** | **AUC** | **AUC 95% CI** | **Calibration MAE** |
| --- | --- | --- | --- | --- |
| Model 1: Main model | LOS + age + infection + previous cerebral infarction history + anemia + cerebral hemorrhage | 0.8635 | 0.837-0.888 | 0.0077 |
| Model 2: Sensitivity model without LOS | age + infection + previous cerebral infarction history + anemia + cerebral hemorrhage | 0.8581 | 0.831-0.885 | 0.01 |

*Note: Calibration was summarized using the mean absolute error (MAE) between predicted and observed event probabilities across deciles of risk. AUC, area under the receiver operating characteristic curve.*

**Supplementary Figure S1. Forest plot of exploratory subgroup analyses of in-hospital acute cerebral infarction risk in patients with gastrointestinal bleeding.***
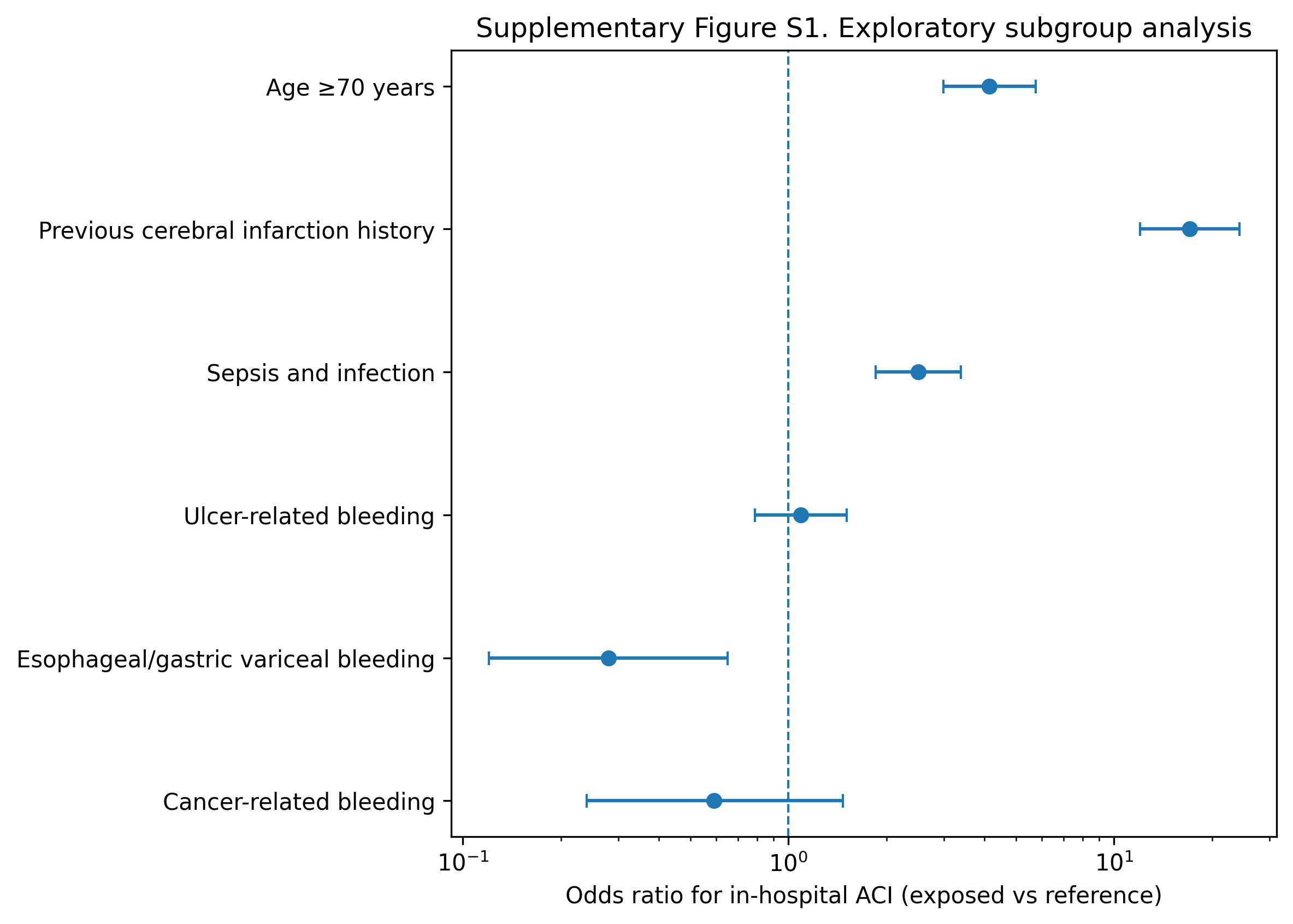
*

*Note:Odds ratios compare the exposed category with the corresponding reference category for each predefined subgroup. Horizontal lines indicate 95% confidence intervals. ACI, acute cerebral infarction; OR, odds ratio; CI, confidence interval.*

**Supplementary Table S3A. Coefficients of alternative models using anemia or hemoglobin level**

| **Model** | **Variable** | **β** | **SE** | **OR (95% CI)** | **P value** |
| --- | --- | --- | --- | --- | --- |
| Model 3: Anemia-based model | Length of hospital stay | 0.0423 | 0.0113 | 1.04 (1.02-1.07) | <0.001 |
| Model 3: Anemia-based model | Age | 0.0485 | 0.0074 | 1.05 (1.03-1.07) | <0.001 |
| Model 3: Anemia-based model | Sepsis and infection | 0.8878 | 0.1779 | 2.43 (1.71-3.44) | <0.001 |
| Model 3: Anemia-based model | Previous cerebral infarction history | 2.6006 | 0.1914 | 13.47 (9.26-19.60) | <0.001 |
| Model 3: Anemia-based model | Anemia | 0.431 | 0.2088 | 1.54 (1.02-2.32) | 0.039 |
| Model 3: Anemia-based model | Cerebral hemorrhage | 1.2475 | 0.5549 | 3.48 (1.17-10.33) | 0.025 |
| Model 4: Hemoglobin-based model | Length of hospital stay | 0.0449 | 0.0112 | 1.05 (1.02-1.07) | <0.001 |
| Model 4: Hemoglobin-based model | Age | 0.0491 | 0.0074 | 1.05 (1.04-1.07) | <0.001 |
| Model 4: Hemoglobin-based model | Sepsis and infection | 0.9136 | 0.1776 | 2.49 (1.76-3.53) | <0.001 |
| Model 4: Hemoglobin-based model | Previous cerebral infarction history | 2.599 | 0.1912 | 13.45 (9.25-19.56) | <0.001 |
| Model 4: Hemoglobin-based model | Hemoglobin level | -0.0005 | 0.0027 | 1.00 (0.99-1.00) | 0.853 |
| Model 4: Hemoglobin-based model | Cerebral hemorrhage | 1.2186 | 0.5618 | 3.38 (1.12-10.17) | 0.030 |

*Note: Model 3 includes the binary anemia variable, whereas Model 4 replaces anemia with continuous hemoglobin level to test the robustness of the association between impaired oxygen-carrying status and in-hospital acute cerebral infarction. β, regression coefficient; SE, standard error; OR, odds ratio; CI, confidence interval.*

**Supplementary Table S3B. Performance comparison between the anemia-based model and the hemoglobin-based model**

| **Model** | **Variables** | **AUC** | **AUC 95% CI** | **Calibration MAE** |
| --- | --- | --- | --- | --- |
| Model 3: Anemia-based model | LOS + age + infection + previous cerebral infarction history + anemia + cerebral hemorrhage | 0.8635 | 0.837-0.888 | 0.0077 |
| Model 4: Hemoglobin-based model | LOS + age + infection + previous cerebral infarction history + hemoglobin + cerebral hemorrhage | 0.8598 | 0.834-0.884 | 0.0087 |

*Note: The two alternative models were compared using discrimination and calibration metrics. AUC, area under the receiver operating characteristic curve; MAE, mean absolute error.*

**Supplementary Table S4A. Exploratory subgroup analysis of in-hospital acute cerebral infarction risk**

| **Subgroup** | **Exposed n** | **Exposed events** | **Exposed incidence** | **Reference n** | **Reference events** | **Reference incidence** | **OR (95% CI)** | **P value** |
| --- | --- | --- | --- | --- | --- | --- | --- | --- |
| Age ≥70 years | 932 | 131 | 14.06% | 1448 | 55 | 3.80% | 4.14 (2.99-5.74) | <0.001 |
| Previous cerebral infarction history | 185 | 84 | 45.41% | 2195 | 102 | 4.65% | 17.07 (12.01-24.24) | <0.001 |
| Sepsis and infection | 878 | 107 | 12.19% | 1502 | 79 | 5.26% | 2.50 (1.85-3.39) | <0.001 |
| Ulcer-related bleeding | 690 | 57 | 8.26% | 1690 | 129 | 7.63% | 1.09 (0.79-1.51) | 0.605 |
| Esophageal/gastric variceal bleeding | 236 | 6 | 2.54% | 2144 | 180 | 8.40% | 0.28 (0.12-0.65) | 0.001 |
| Cancer-related bleeding | 103 | 5 | 4.85% | 2277 | 181 | 7.95% | 0.59 (0.24-1.47) | 0.252 |

*Note: For each subgroup, the “exposed” category was compared with its corresponding reference category using unadjusted event counts. Odds ratios were calculated from 2×2 contingency tables; Fisher’s exact test was used when appropriate. CI, confidence interval.*

**Supplementary Table S4B. Main-model discrimination within exploratory subgroups**

| **Subgroup** | **Category** | **n** | **ACI events** | **Incidence** | **Main model AUC** |
| --- | --- | --- | --- | --- | --- |
| Age ≥70 years | Reference | 1448 | 55 | 3.80% | 0.891 |
| Age ≥70 years | Exposed | 932 | 131 | 14.06% | 0.775 |
| Previous cerebral infarction history | Reference | 2195 | 102 | 4.65% | 0.806 |
| Previous cerebral infarction history | Exposed | 185 | 84 | 45.41% | 0.562 |
| Sepsis and infection | Reference | 1502 | 79 | 5.26% | 0.876 |
| Sepsis and infection | Exposed | 878 | 107 | 12.19% | 0.81 |
| Ulcer-related bleeding | Reference | 1690 | 129 | 7.63% | 0.868 |
| Ulcer-related bleeding | Exposed | 690 | 57 | 8.26% | 0.854 |
| Esophageal/gastric variceal bleeding | Reference | 2144 | 180 | 8.40% | 0.859 |
| Esophageal/gastric variceal bleeding | Exposed | 236 | 6 | 2.54% | 0.904 |
| Cancer-related bleeding | Reference | 2277 | 181 | 7.95% | 0.868 |
| Cancer-related bleeding | Exposed | 103 | 5 | 4.85% | 0.816 |

*Note: AUC values were calculated by applying the primary multivariable model to each subgroup separately. These analyses are exploratory and intended to describe subgroup-specific model discrimination rather than to establish formal interaction effects. AUC, area under the receiver operating characteristic curve; ACI, acute cerebral infarction.*
